# Review of an Article Entitled Dental Hygiene
*This Article was published in the August Number of *Journal*, copied from the Dental Times."


**Published:** 1871-01

**Authors:** S. P. Cutler


### ARTICLE II.

### *Review of an Article Entitled Dental Hygiene.**

By Prof. S. P. Cutler, M. D., D. D. S.

(Read before the New Orleans Dental Association.)

This singular production, notwithstanding its flowing and elegant diction, has some feeble and weak points according to scientific dicta.

On page 183, the author says "The capillary net work which derives its nourishment from the systemic circulation, constitutes the source of vitality to the teeth, and to keep it in an active healthy condition so as to perform its functions and build up and establish those hard and unyielding structures &c."

This capillary net work spoken of above, as I understand

*This Article was published in the August Number of *Journal*, copied from the "Dental Times."

the subject, constitutes the connecting link between the veins and arteries, a part or parcel of the systemic circulation, and constitutes the chief points of nutrition though taking place outside of the flowing tide.

Same page says, "The human teeth are of that class of organs which are not in direct relation with the blood vessels, and yet which derive their whole nutriment and the material of their functional operations from the blood. In all respects they are a separate and distinct creation."

What an incongruity there is in the above statement He does not seem to regard the highly neural and vascular pulps with their vessels and nerves coming into their interior direct from the dental arteries and nerves entering into the apices of fangs, as constituting any direct connection with the blood vessels. I certainly shall differ with him as I regard the connection intimate to the fullest extent of the term.

I have been led to believe that the teeth unite or combine in themselves the three great systems, so far as they go, that is dermal and neuro-hermal in the highest state of perfective development, equally as much so as the skin, with this main difference that the external coat of the teeth, the enamel, does not exfoliate like the epidermis, owing to its extreme hardness; in all other respects they are homalogous.

On page 184, the writer says, "speaking of capillary nutrition in tissues and the complex organism, nature and structure of teeth being such as to deprive them of recuperative action, stagnation leading to decompositions and decays are the result."

Now if stagnation in these tissues leads to decays and decompositions, why do we not find a similar result taking place in the bones themselves throughout the entire organism; they being very similar in compositions, only less dense and always being covered up, thereby precluding the posbiility of local decays, except in cases of necrosis, like the teeth, in consequence leading to the conclusion that teeth decay

*de loco*, mainly independent of systemic influences so far as decays *per se* are concerned.

I do not wish to be understood as advancing the idea that all teeth are equally well developed and capable of the same resistance to external influences by any means.

Page 186 speaking of the three tissues composing the teeth says, "And arrangement gives the cementum, the position nearest the vascular tissues, because of its better adaptation to hold the fluid thrown from the blood to be imbibed by the capilaries of the more highly organized structure of the dentine.

In speaking of the dentine I am unable to comprehend his meaning unless he refers to the tubuli.

Must we look to the cemental membrane for nutrition to the dentine, if so how are we to account for the interior life and sensibility of the whole tooth whe the fang has been denuded of its periosteum nearly to the apex for years, which we oftenwitness. But when we look to the blood vessels and nerves entering the interior of the tooth at apex of the fang, we have no longer any difficulty so long as they remain intact in accounting for this sensibility or vital manifestation.

The periosteal membrane, which the writer assigns to such extraordinary functions, cannot, in my opinion, be considered or regarded as anything higher than ordinary periosteum, holding only a low office in the economy as furnishing nutrition to the cement in part and to the alveolus, and a bond of union or connection between jaw bone and teeth, not confirming sensorial vitality to any extent to the dentine, if to the cement only in part or limited extent.

Same page, writer says: "The dentine is perhaps the most delicately organized fabric within the entire range of the economy." "The capillary vessels contain a fluid composed of the finest particles of the body."

The writer does not mention the subject of tubular fibrils, only a fluid occupancy, thereby still adhering to Kolliker's old fluid hypothesis.

On page 187 He speaks of articular cartilage vessels in connection with capillary."

It is well known that cartilages as spoken of above, have no circulation, as they have neither vessels or nerves, only osmotic for nutrition.

The articular are the most perfect and complete of all the cartilages in the organism.

Page 188 says, "Irregularities in the capillary circulation have been noted when the systemic circulation was in a perfect condition."

The writer speaks of the capillary circulation as independent of systemic to some extent, as though the capillary did not form a part and parcel, and the most essentially vital too of the systemic circulation. Then how could the one be disturbed, and at the same time the other, not only where the local disturbance is exceedingly limited, and where the circulation could easily pass around the disturbed point withany serious disturbance; this could not well apply to the disturbance in a tooth to any considerable extent without producing more or less general disturbance.

Page 180 says " the liability of teeth to decay through an undue acceleration of local or capillary circulation from which there is a gorging or choking of the tissues, thereby interupting functional power and producing results similar to an enfeebling insufficiency of capillary nutrition." After a tooth is once fully developed it matters but little whether or not there is a due or undue amount of circulation in the capillaries of tooth pulp; so long as the mouth is not in an acid condition there will be no decays, the circulation carried on in the interior of a tooth having no sensible effect within a limited time on the condition of the dentine. In this statement I am not denying the fact that slow and gradual changes do go on in the dentine from youth to old age gradually becoming more earthy and closed and less vital, even in some instances perhaps of diminution in density of dentine, consequently of earthy matters, rendering the teeth less resistant to local influences the cause of decays.

Page 191 says, "In the softer tissues these pathological conditions can be arrested before inflammation or stagnation sets in but in the denser or bony tissues and especially those forming the teeth the remedy cannot be applied; interstitial deposit cannot be corrected or reduced to a normal standard and a stagnation is the result which finally produces disorganization and decay.

Now if decays of teeth depend on internal causes alone, we certainly would have no knowledge of a decayed condition of a tooth until the outside should fall in leaving the nerve exposed, and no chance for filling only on an exposed nerve, and who has ever seen this character of decay? I can safely say no one.

Now that changes are constantly taking place or going on in teeth, as in all other living animal structures, I am ready to admit, as such is a recognized fact.

There is no such thing as standing still any where in the universe, all things are in motion and undergoing change.

That there is different degrees of density in teeth of different individuals I am fully aware, and that teeth containing a minimum amount of earthy salts more readily decay, other things equal, I am also fully aware and *vice versa.*

That something like *mollities ossium* which might with propriety be denominated *mollities dentium* sometimes takes place in individual cases, I have no doubt, which may depend on systemic causes such as an acid diathesis, if not pervading the entire organism at least the regions about the teeth, removing the lime salts to such an extent that the teeth decay in a most rapid manner; in all such cases we may expect to find an acid condition of the mouth. In this as in all other forms of decay, an acid is the factor employed in the work; I might add that all such decays are of the albous or white variety with few if any exceptions.

In such cases the bones themselve may or may not be seriously involved, owing to the fact of the condition being general or local in its character.

In saying this much I am assuming a little, as the data at present extant is somewhat limited. There are other cases of softening of teeth or rather destruction from the use of elixir of vitriol and muriated tincture of iron, where the surfaces of the enamel first and subsequently the dentine, is extensively softened and decayed away with characteristic appearances familiar to all dentists of long experience.

In such cases where the remedy has been long continued even an acid diathesis might be anticipated sufficient to co-operate in the local distinction, the bones even in some instances may be temporarily implicated but subject to subsequent reparation.

So even in case of the teeth after the cause of the softening is removed, subsequent hardening may and does take place if the process be not too long continued and the destruction too extensive, especially if from constitutional causes.

